# Medical Report of the House of Recovery and Fever Hospital, for the Year 1827

**Published:** 1829-07-01

**Authors:** 


**FEVER HOSPITAL, CORK STREET.**

MEDICAL REPORT OF THE HOUSE OF RECOVERY, AND FEVER-HOSPITAL, CORK-STREET, DUBLIN, FOR THE YEAR, 1827. By JOHN O'REARDON, M. D.

The importance of such reports as these from public institutions is now sufficiently appreciated, and Dublin has long set a good example to this metropolis—an example which, we grieve to say, has not been followed. Why do not the talented medical officers of our own FEVER-HOSPITAL present their brethren with the results of their observations, in the form of annual MEDICAL reports? We hope this hint will stimulate the physicians of that institution to the undertaking.

Dr. O'Reardon treated, in the year 1827, eight hundred and three patients with fever in the hospital. He calculated that about six sevenths of the continued fever cases were complicated with various degrees of visceral affection—namely, with sub-inflammatory ailments of the head, lungs, fauces, stomach or intestines, or with rheumatism. In some cases, only one organ was affected materially—in many cases, two or even three organs were cotemporaneously inflamed in a greater or lesser degree.

"In the fully formed common Fever of these countries we observe an elevated temperature of body, acceleration of pulse, some degree of headach; foulness or rawness of tongue, not unfrequently enlargment of this organ, sometimes thickness of it, at other times a parched, glossy, or a dry rough chopped state of it, resembling a thinly incrusted blood-oozing sore; face either flushed or of a soiled dusky aspect, and of a semi-bloated appearance in the early stage of Fever; respiration somewhat accelerated and oppressed; nausea, thirst, costiveness; epigastrium full, and over sensible to the tact; liability to general abdominal soreness and inflammation: aching of various parts of the body, namely of the spine, joints, thighs, and arms; a sense of general lassitude and heaviness: a state either of restlessness or of somnolency. Such a condition assuredly belongs to a general internal subinflammatory action of the system, an action like all inflammatory movements, mainly depending on and kept up by an alteration and derangement of the circulation. When Fever is high the general subinflammatory movement in question often assumes a more decidedly inflammatory turn; and if it be not complicated from the beginning, in the progress of the great majority of cases it becomes extended to one two or more of the organs of the head, thorax, and abdomen, or to the muscular fibres, their membranes and tendinous aponeuroses, so as as to cause rheumatism.

"Combined with the general subinflammatory, or inflammatory state in question, there usually is a nervous condition, which modifies the Fever in some degree.—We likewise often observe symptoms of vitiated bile and disordered functions of the liver and stomach, probably arising from the encreased temperature of the body, and the altered state of the circulation, and reacting on the Fever so as to produce corresponding modifications thereof. Many of our patients are seized at the same time with general pyrexia and visceral inflammation. In some other instances the local disorder is the primary complaint, attended with what is commonly termed Symptomatic Fever. The former course of attack belongs to the genus which is the leading subject of this paper, viz. to general continued Fever, embracing its complications. The latter might in like manner be classed as a species of the same genus.

"I conceive that in a nosological table, as well as in the mind of the practising physician, the Febres and Phlegmasiæ should belong to one order.

"It is often difficult, and not always necessary, to ascertain exactly whether the priority of invasion belongs to the general Fever, or to the visceral affection. Some Fevers commence with symptoms of sanguineous plethora or congestion in the head; some with a deranged condition of the stomach, and especially of its mucous membrane and glands, and of the continuation of said membrane to the fauces and tongue. The stomach seldom fails to sympathize strongly with the head, when the latter suffers from severe fixed Cephalœa, caused by vascular turgescence or inflammation.

"Some Fevers begin with, or are from the commencement attended by, either sub-inflammation or complete inflammation of a part or the entire of the thoracic viscera; others with inflammation of the liver or bowels, or of some other part in the abdomen: others again with rheumatism. Whether the Complicatæ be simultaneous with or secondary or anterior to the general Fever, the ratio medendi is nearly or entirely the same. It is, however, quite essential in practice to ascertain as well as possible the organ or organs principally diseased; as by the early employment of the most appropriate means for cure thereof, we shall be the better enabled to preserve our patients' lives, and abridge the duration of their Fevers."

Our author considers all the divisions of fevers in our classic medical works, on which so much Greek and Latin has been expended in christening them by desperate long and hard names, "as mere varieties of one genus—that is, of the above-described general subinflammatory condition, constituting fever." These varieties, he thinks, are produced by diet, occupation, locality, temperament, habit of body, state of mind, and many other accidental circumstances, together with climate, season, treatment—and epidemic influence.

"The above view of Fever, which in my mind is that of nature, removes that obscurity and confusion which long prevailed, and in some places still exists in the management of Fever. Though comprehensive, it does not confound any diseases which are naturally separate. It admits and requires the utmost attention in distinguishing the varieties and successive changes of Fever, and in discriminating the character of each disordered viscus. It indicates a rational, clear, effective, and tolerably uniform line of practice in the treatment of Fevers, and their manifold complications—a practice which the best modern physicians are for these many years back gradually adopting, as if it were by common consent.

"This practice consists mainly in the Antiphlogistic plan of treatment, modified and varied according to circumstances, and especially according to the character, grade, and period of the Fever, its complications, and the patient's pulse and strength.

"Bilious and hepatic symptoms are to be subdued by well known appropriate remedies. These are remedies which, so far from being opposed to, are for the greater part in unison with the fore-mentioned Antiphlogistic plan. This plan based, it is presumed, on sound principles, is extensive and varied in its application. It admits of the mildest practice in mild cases, as well as of the most active and efficient treatment in severe cases. It is suitable not only to the diseases above-mentioned, but it is also applicable to the exanthemata or eruptive fevers, which require to be treated pretty much on the same principle with common Fever and its modifications: but in the exanthemata we must be cautious to prevent the disappearance of the eruption before the proper period; and we must be ready to obviate or remedy visceral determinations.

"In the characteristic sketch of common Fever, above drawn, in page 10, it has not been my object to trace its phenomena on regularly to its decline and termination. But I may here add that whatever may be its appearance in the beginning, and by whatsoever title it might be denominated by Nosologists, whether by that of Synocha, Synochus, or Typhus; in its advanced state it becomes occasionally marked by adynamic spasmodic nervous symptoms, with a black or blackish tongue, and a very peculiar alteration of countenance; symptoms which supervene on or take place of the acute sub-inflammatory or inflammatory stages. Under these circumstances other indications arise, which call forth the application of the whole medical mind. The Methodus Antiphlogistica, is to be employed not only in a very mitigated manner, but it must be combined with, and often superseded by antispasmodic, antinervous, diffusively stimulating and tonic remedies. As in the class of tonic medicines, cinchona holds a prominent station, I think it right to observe that this article is scarcely ever useful, and is often injurious in continued Fever during the existence of a hot skin, a quick pulse, and a loaded tongue; cases of gangrene excepted, where indeed the temperature is in general any thing but high, and where cinchona, aided by camphorated mixture, good wine, and a suitable position of the body, is an extremely valuable curative agent. I have already noticed the great efficacy of this bark in Erysipelas, after the previous substraction of some blood from the arm."

Agues have not been very frequent or troublesome in Dublin. The following quotation contains a rather curious opinion.

"I formerly had an opportunity of witnessing multitudes of Agues of all types in the great hospitals La Salpetriere, and L'Hotel Dieu in Paris. I was a pupil in the latter establishment on the return from Walcheren of the French and some Polish sick soldiers; many of whom were as violently afflicted with the Walcheren intermittents as our troops who succeeded them in that unhealthy island soon afterwards were. The result of my experience is a conviction that Ague is a nervous and spasmodic malady of a very peculiar nature, and that its place in nosological tables belongs to the class of Neuroses and the order of Spasmi. The involuntary, unrestrainable, and extraordinary shaking of the whole frame; the sort of spasmodic closing of each hand, and drawing in and upwards of the arms and lower extremities, and their alternate stretchings; the particular inward sensation in the region of the epigastric ganglions and plexuses during the rigor of the intermittent fit: the flushed countenance, the cephalœa, the heaviness, drowsiness, and sometimes stupor and even apoplectic sopor and stertor at the close of the paroxysm; the occasional influence of emotions of the mind in producing or stopping intermittent paroxysms; the periodical visitations of Ague, the general freedom from complaint in the intervals; the liability of the disease to frequent returns after its cure; its long continuance not only for months, but, in some instances, for a great number of years, such as from four to seven, and even so far as twenty-six years, or during life, of which my clinical case books contain detailed histories:—These symptoms and circumstances seem to me to denote a very obvious analogy between Ague and other convulsive maladies, especially Epilepsia and Hysteria; an analogy rendered still closer by the notable similarity or sameness of the tonic and antispasmodic remedies prescribed in the first and latter affections. Due attention to this analogy may perhaps, ere long, lead physicians to the discovery of either a permanent cure, or a meliorated treatment for Epilepsy."

Of the reality of febrile contagion our author is firmly convinced.

"It is well known to all physicians who attentively mark the frequent instances there are of many of the poor becoming sick successively in the same house and in one room. If, out of five or six members of a badly-lodged and ill-clad indigent family, necessitated to sleep together, one be seized with Fever, the person who lies closest to him and faces him, will receive and inhale his heated, irritating, and morbific breath, and imbibe his perspirable matter, and will sicken. The remainder of the family will in like manner become successively diseased. — Should one of these individuals, who may happen to be, unknowingly, in the commencement of Fever, quit the sick-room, visit relatives or friends elsewhere, and sleep with them, his fever will soon develope itself, and be communicated to his new bed-fellows. To this manifest source of contagion may be added the circumstance of the frequent migrations from one wretched lodging to another, of indigent families, who not unfrequently carry sickness in their train, or meet with it in their new places of abode."

---

